# Neurosurgical Resection for De Novo Brain Metastases from Non-Small Cell Lung Cancer: Real-World Evidence for Multidisciplinary Decision-Making

**DOI:** 10.3390/jcm15186955

**Published:** 2026-09-08

**Authors:** Xinyu Wang, Hongliang Mao, Fengchun Mu, Chen Yang, Lin Cheng, Wenxi Huang, Yongchang He, Yujia Chen, Hongqing Cai, Qi Liu, Ke Hui, Ke Hu, Liang Zhang, Jinghai Wan, Ming Yang

**Affiliations:** Department of Neurosurgery, National Cancer Center/National Clinical Research Center for Cancer/Cancer Hospital, Chinese Academy of Medical Sciences and Peking Union Medical College, Beijing 100021, China

**Keywords:** brain metastases, non-small cell lung cancer, de novo brain metastases, surgical resection, precision oncology

## Abstract

**Background:** Patients presenting with concurrent lung and brain lesions pose a unique diagnostic and therapeutic challenge. The role of neurosurgical resection in the management of de novo non-small-cell lung cancer brain metastases (NSCLC-BM) remains poorly defined. **Methods:** Consecutive patients undergoing resection for NSCLC-BM between February 2018 and October 2025 were retrospectively analyzed. De novo NSCLC-BM was defined as radiographic detection of pulmonary and intracranial lesions concurrently or within 1 month during the initial diagnostic work-up. Clinical characteristics and outcomes were compared between de novo and metachronous brain metastases (BM). Multivariable Cox regression identified factors associated with overall survival (OS). Factors associated with long-term postoperative survival in the de novo subgroup were further explored using Firth logistic regression. **Results:** Among 248 surgically treated patients, 113 (45.6%) presented with de novo BM. Compared with metachronous BM, de novo patients more frequently presented with symptomatic and multifocal intracranial disease. Among 73 de novo patients with evaluable survival, median postoperative OS was 57.0 months (95% CI, 22.9–not reached). Postoperative OS and intracranial progression-free survival (iPFS) did not differ significantly between the two groups, and de novo presentation was not independently associated with postoperative survival. Driver-positive status and lower intracranial tumor burden were associated with durable postoperative survival in the de novo subgroup. **Conclusions:** Neurosurgical resection represents a reasonable option for appropriately selected patients with de novo NSCLC-BM. These findings provide real-world evidence to inform multidisciplinary, individualized decision-making in patients presenting with concurrent lung and brain lesions.

## 1. Introduction

Patients presenting with concurrent lung and brain lesions represent a distinct neurosurgical scenario [1,2]. Unlike metachronous brain metastases (BM), which are typically detected during surveillance or systemic treatment follow-up, de novo BM often presents before histology or molecular status has been established, while neurological symptoms or mass effect may require timely intracranial intervention [3,4]. Clinicians must therefore determine both the diagnostic tissue source and treatment sequence, choosing among thoracic biopsy, intracranial biopsy, or resection of a symptomatic or dominant brain lesion [5,6]. In selected patients, surgical resection may be clinically advantageous because it can simultaneously provide intracranial disease control, histological diagnosis, and tissue for molecular profiling [7].

BM at initial lung cancer presentation has traditionally raised concerns regarding limited survival and uncertain benefit from aggressive local therapy [8]. However, this assumption requires reconsideration in the modern treatment era [9]. CNS-active targeted therapies, particularly newer-generation EGFR and ALK tyrosine kinase inhibitors with improved intracranial activity, have substantially prolonged disease control in molecularly selected non-small-cell lung cancer brain metastases (NSCLC-BM) patients [10,11,12,13,14,15]. Along with immunotherapy, stereotactic radiotherapy, and multidisciplinary care, these advances have made durable survival increasingly achievable in selected patients with BM [16,17]. Existing studies of de novo or synchronous NSCLC-BM have largely focused on systemic treatment outcomes or thoracic surgical strategies, whereas the contemporary role of neurosurgical resection in de novo NSCLC-BM requires reappraisal [8,18].

To better define the role of neurosurgical resection in patients presenting with de novo NSCLC-BM, we retrospectively evaluated postoperative outcomes, compared them with those of patients with metachronous BM, and explored clinical factors associated with durable survival.

## 2. Methods

### 2.1. Study Design and Patient Selection

This retrospective cohort study reviewed consecutive patients who underwent surgical resection for pathologically confirmed NSCLC-BM at the Department of Neurosurgery, National Cancer Center/Cancer Hospital, Chinese Academy of Medical Sciences and Peking Union Medical College between February 2018 and October 2025.

**Figure 1:**
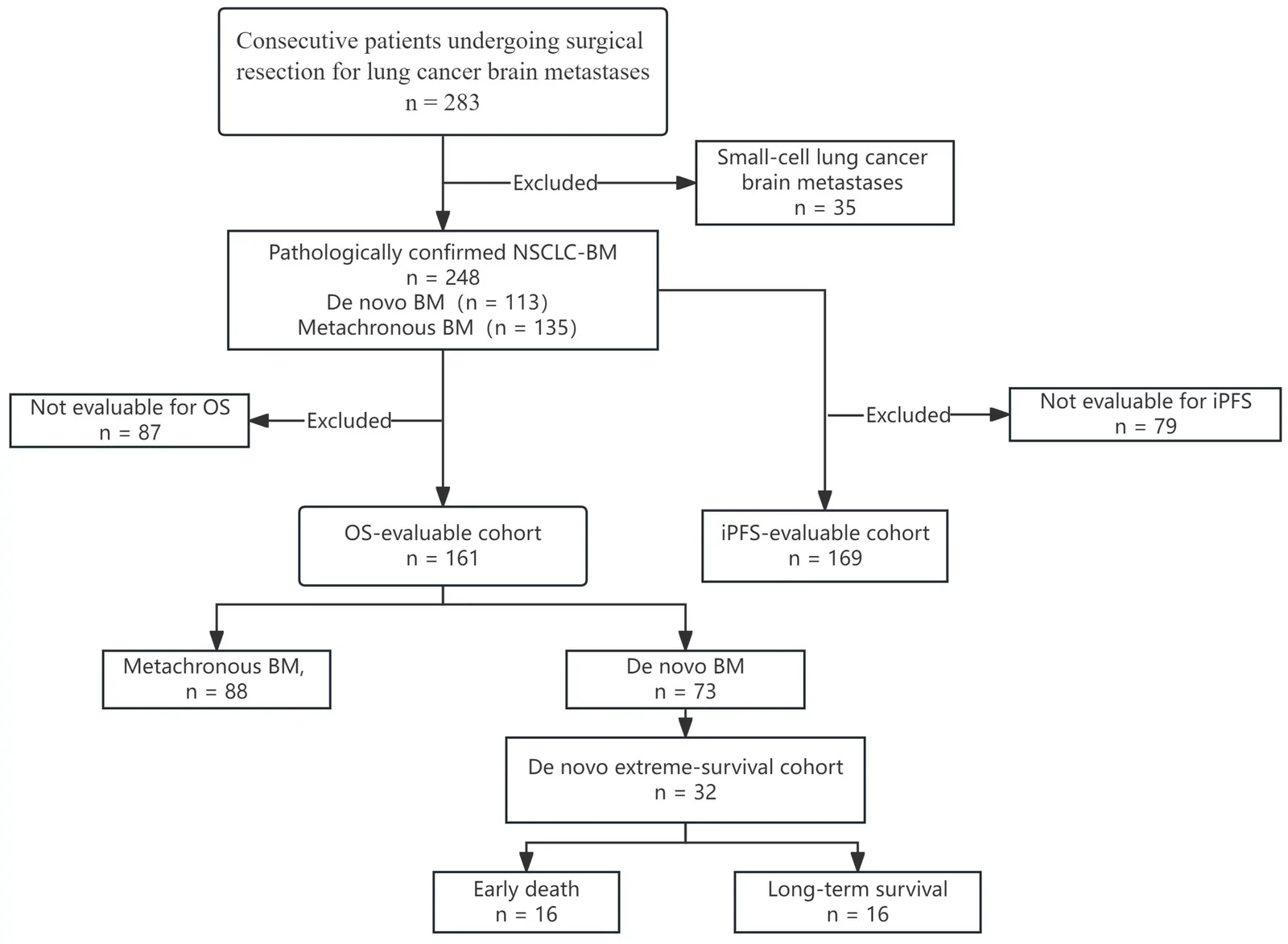
Study flow diagram. (Flowchart of patient selection and analytic cohorts. Of 248 patients with pathologically confirmed NSCLC-BM, 161 were evaluable for OS and 169 for iPFS; non-evaluability was primarily attributable to incomplete follow-up. The exploratory extreme-survival analysis included 32 patients with de novo BM and classifiable outcomes (OS < 12 months or ≥36 months)).

Patients with small-cell lung cancer brain metastases were excluded. The final study cohort included patients with pathologically confirmed NSCLC-BM. Patients with known BM timing were included in the baseline comparison between de novo and metachronous BM. The study flow is summarized in Figure 1.

The study was approved by the Ethics Committee of National Cancer Center/Cancer Hospital, Chinese Academy of Medical Sciences and Peking Union Medical College (approval No. 26/049-5774). The requirement for informed consent was waived because of the retrospective design. The study was conducted in accordance with the Declaration of Helsinki.

### 2.2. Definition of De Novo and Metachronous BM

De novo NSCLC-BM was defined as radiographic detection of pulmonary and intracranial lesions concurrently or within 1 month of each other during the initial diagnostic work-up. The 1-month window allowed for short differences in the timing of chest and brain imaging within the same diagnostic episode. Metachronous BM was defined as BM diagnosed more than 1 month after the initial diagnosis of NSCLC. No metachronous cases were clustered near this cutoff, with the shortest interval between the initial NSCLC diagnosis and BM diagnosis being 56 days.

### 2.3. Clinical and Molecular Variables

Demographic, clinical, radiographic, molecular, and treatment-related variables were extracted from electronic medical records, including age, sex, smoking history, recent weight loss, preoperative functional status, histology, driver gene status, intracranial tumor burden, extracranial disease status, extent of resection, and postoperative therapies. Intracranial tumor burden was characterized by the number of BM, maximum BM diameter, and total BM volume. Combined intracranial–extracranial disease extent was classified as single BM only, single BM with extracranial systemic metastasis, or multiple BM. Patients with multiple BM were grouped as multiple BM regardless of extracranial disease status. Symptomatic BM was defined as the presence of neurological symptoms before surgery.

Driver status was classified as positive, negative, or unknown. Driver-positive disease was defined as the presence of an oncogenic driver alteration (e.g., EGFR, ALK, ROS1, KRAS, BRAF V600E, MET exon 14 skipping, RET, or NTRK). Driver-negative disease indicated no detected driver alteration among tested genes, whereas patients without molecular testing were classified as driver-unknown. Given the heterogeneity in therapeutic actionability, particularly among KRAS variants, sensitivity analyses used a variant-level actionability classification, with only alterations with available matched targeted therapies considered actionable; among KRAS variants, only G12C was classified as actionable.

### 2.4. Outcomes

The primary endpoint was overall survival (OS), defined as the interval from BM surgery to death from any cause or last follow-up. The secondary endpoint was intracranial progression-free survival (iPFS), defined as the interval from BM surgery to intracranial progression or death, whichever occurred first. Intracranial progression was determined from follow-up neuroimaging and clinical records.

Patients were considered evaluable for OS if postoperative vital status could be reliably ascertained, and for iPFS if sufficient clinical and/or neuroimaging follow-up was available to determine intracranial progression or death. Patients for whom the respective endpoint could not be reliably ascertained, primarily because of loss to follow-up, were excluded from the corresponding analysis.

In the de novo subgroup, exploratory extreme-survival groups were defined to identify early postoperative failure and long-term survival. The early-death group included patients who died within 12 months after BM surgery, whereas the long-survival group included patients who survived for at least 36 months. Patients with intermediate survival or immature censored follow-up were excluded from this extreme-survival comparison.

### 2.5. Statistical Analysis

Continuous variables were summarized as medians with interquartile ranges and compared using Wilcoxon rank-sum tests. Categorical variables were summarized as counts with percentages and compared using Fisher exact tests. Survival curves were estimated using the Kaplan–Meier method and compared using log-rank tests.

Multivariable Cox proportional hazards regression was used to identify factors associated with OS. The main adjusted model included BM timing, driver status, maximum BM diameter, combined intracranial–extracranial disease extent, and weight loss. Sensitivity models additionally adjusted for extent of resection or symptomatic BM presentation. Variables were selected based on clinical relevance and avoidance of excessive collinearity.

In the de novo extreme-survival analysis, Firth penalized logistic regression was used to reduce small-sample bias and address separation, because no driver-negative patient achieved long survival. For this analysis, driver status was dichotomized as driver-positive versus driver-negative, and patients with unknown driver status were excluded from the model. Odds ratios greater than 1 indicated increased odds of long survival. A sensitivity Firth model replaced maximum BM diameter with log-transformed total BM volume. Two-sided *p* values < 0.05 were considered statistically significant. Analyses were performed using R version 4.5.2.

## 3. Results

### 3.1. Patient Cohort

A total of 248 surgically treated patients with NSCLC-BM had known BM timing and were included in the baseline timing comparison. Among them, 113 patients had de novo BM and 135 had metachronous BM. The OS-evaluable timing cohort included 161 patients, including 73 with de novo BM and 88 with metachronous BM. The iPFS-evaluable cohort included 169 patients, including 77 with de novo BM and 92 with metachronous BM. Median OS in the OS-evaluable cohort was 57.0 months (95% CI, 44.4–not reached), with 1- and 2-year OS rates of 81.3% and 63.4%, respectively. Median iPFS in the iPFS-evaluable cohort was 13.7 months (95% CI, 11.4–18.0).

### 3.2. Clinical Profile of Patients with De Novo BM

Baseline characteristics according to BM timing are summarized in Table 1. Compared with metachronous BM patients, de novo BM patients were more frequently symptomatic at presentation (92.9% vs. 72.6%, *p* < 0.001) and more often current smokers (39.8% vs. 8.2%, *p* < 0.001).

De novo BM was associated with a more extensive intracranial disease burden, including a higher number of BM, more frequent multiple BM, and combined supra- and infratentorial involvement (all *p* < 0.001), whereas maximum BM diameter and total BM volume were similar between groups. Patients with de novo BM also had less favorable overall disease and surgical profiles, with more extracranial disease, lower GTR rates, and poorer GPA scores (all *p* ≤ 0.021).

The prevalence of oncogenic driver alterations was comparable between groups. The frequency of driver-positive disease did not differ significantly (62.8% vs. 63.7%), although driver status was more frequently unknown in the de novo group (13.3% vs. 3.0%), contributing to an overall difference in driver-status distribution (*p* = 0.005). Similar findings were observed in the OS-evaluable cohort (Supplementary Table S1).

### 3.3. De Novo BM Was Not Associated with Inferior Postoperative Survival

**Figure 2:**
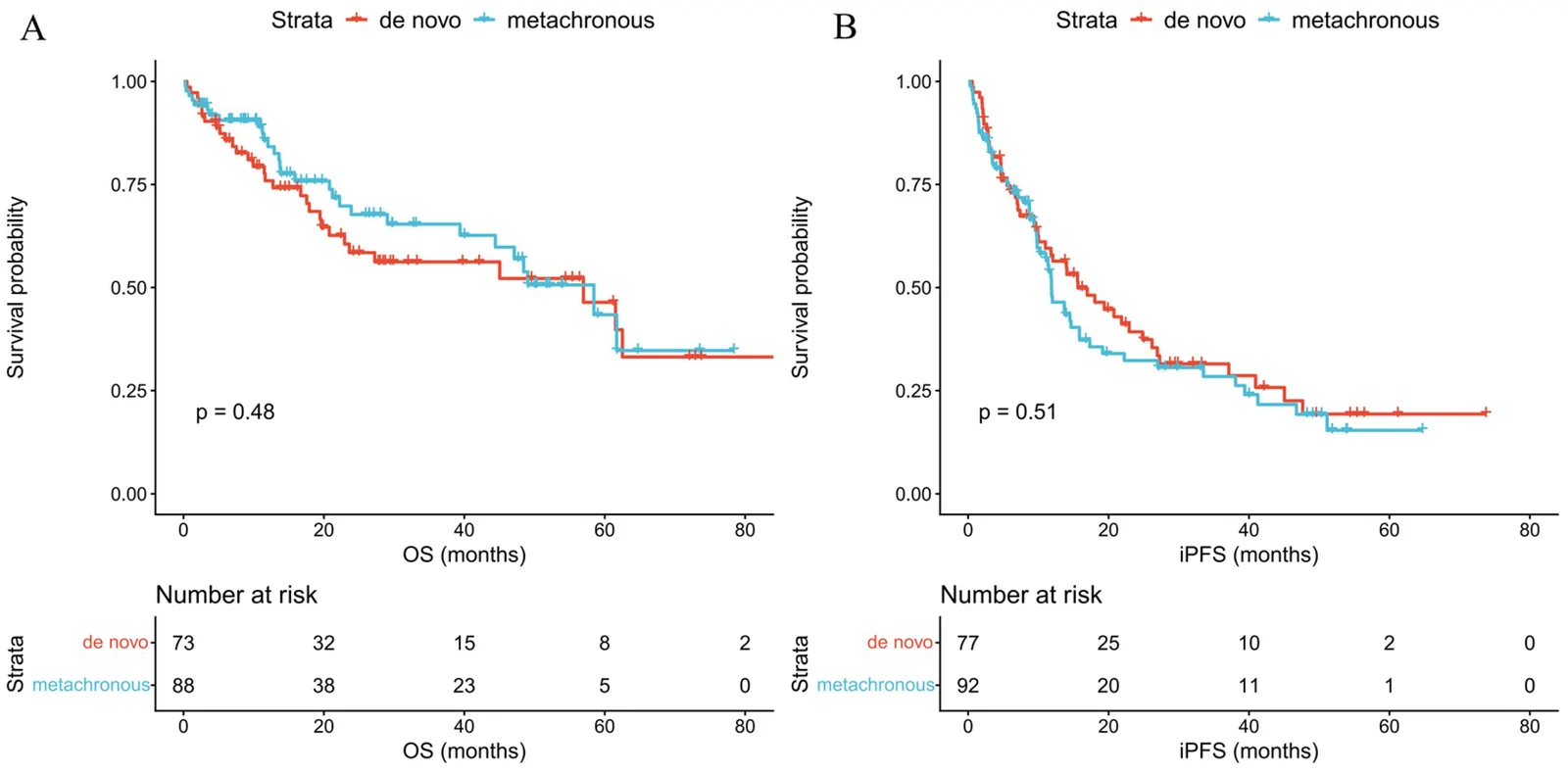
Kaplan–Meier analysis of postoperative survival according to BM timing. (**A**) Overall survival (OS) after brain metastasis resection in surgically treated patients with de novo and metachronous NSCLC-BM. (**B**) Intracranial progression-free survival (iPFS) according to BM timing. (Survival was calculated from the date of brain metastasis surgery. *p* values were calculated using the log-rank test. Tick marks indicate censored observations. Abbreviations: BM, brain metastasis; iPFS, intracranial progression-free survival; NSCLC, non-small-cell lung cancer; OS, overall survival.).

Despite their more symptomatic and complex baseline disease profile, de novo BM patients did not have significantly worse postoperative survival outcomes than metachronous BM patients. In the OS-evaluable cohort, Kaplan–Meier analysis showed no significant difference in OS between de novo and metachronous BM patients (log-rank *p* = 0.48; Figure 2A). Similarly, iPFS did not differ significantly between groups in the iPFS-evaluable cohort (log-rank *p* = 0.51; Figure 2B).

**Figure 3:**
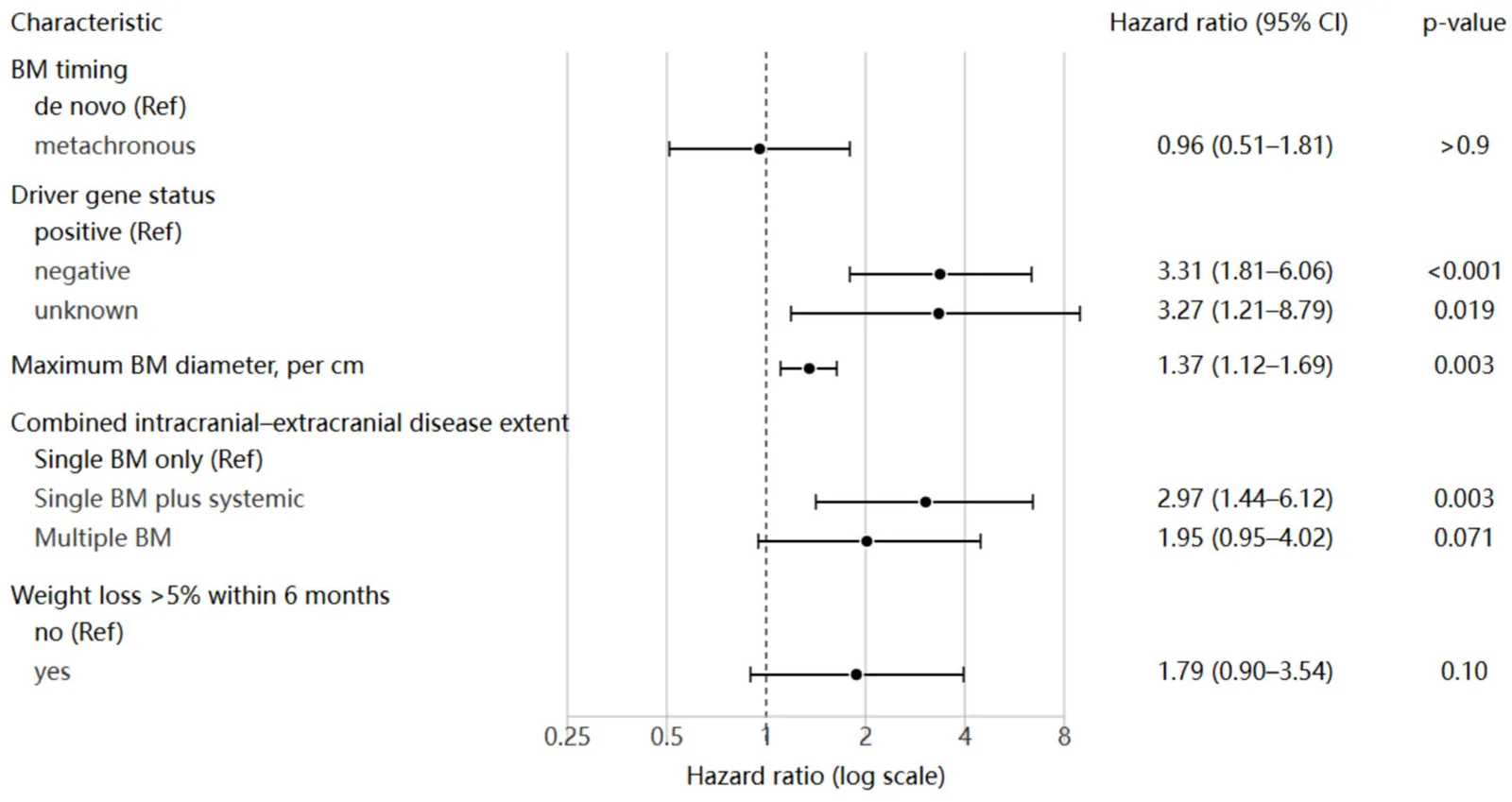
Forest plot of multivariable Cox regression analysis for overall survival in patients with surgically treated NSCLC brain metastases. (Hazard ratios (HRs) and 95% confidence intervals (CIs) are shown for variables included in the multivariable Cox proportional hazards model. De novo presentation and driver gene-positive status were used as reference categories. HRs greater than 1 indicate higher hazard of death.).

In the multivariable Cox model adjusted for driver gene status, maximum BM diameter, combined intracranial–extracranial disease extent, and recent weight loss, BM timing was not independently associated with OS (Table 2). The corresponding hazard ratios and 95% confidence intervals are illustrated in Figure 3. Compared with de novo BM, metachronous BM did not show a significant OS advantage (HR, 0.96; 95% CI, 0.51–1.81; *p* > 0.9). Independent adverse factors included driver-negative disease (HR, 3.31; 95% CI, 1.81–6.06; *p* < 0.001), driver-unknown status (HR, 3.27; 95% CI, 1.21–8.79; *p* = 0.019), larger maximum BM diameter (HR per 1 cm increase, 1.37; 95% CI, 1.12–1.69; *p* = 0.003), and single BM with extracranial systemic metastasis (HR, 2.97; 95% CI, 1.44–6.12; *p* = 0.003). Multiple BM showed an adverse but non-significant association with OS (HR, 1.95; 95% CI, 0.95–4.02; *p* = 0.071). Recent weight loss also showed a non-significant adverse trend (HR, 1.79; 95% CI, 0.90–3.54; *p* = 0.10).

Sensitivity Cox models adjusting for extent of resection or symptomatic BM presentation yielded consistent results, with BM timing remaining non-significant. These findings suggest that de novo presentation itself was not a marker of inferior postoperative survival in surgically selected NSCLC-BM patients.

### 3.4. Characteristics of De Novo BM with Long-Term Survival

**Figure 4:**
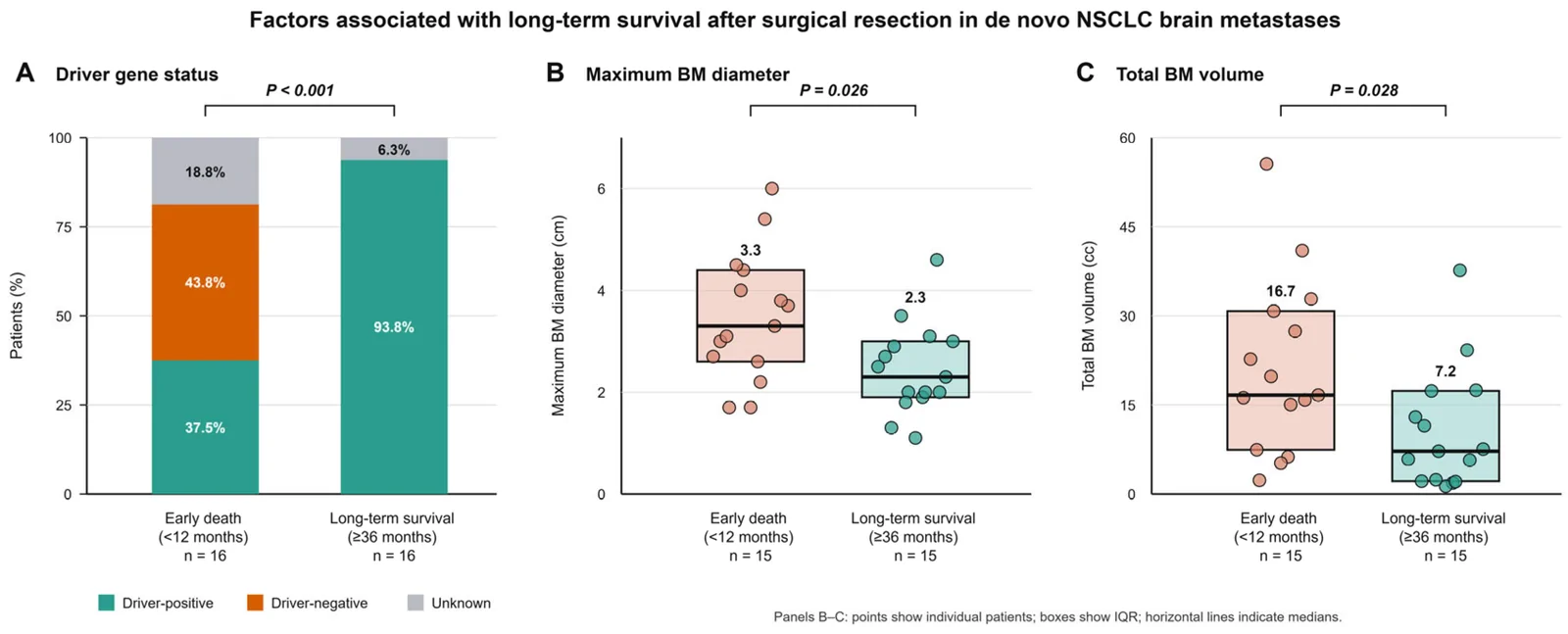
Factors associated with long-term survival after surgical resection in de novo NSCLC brain metastases. (**A**) Distribution of driver gene status among patients with early death (<12 months) and long-term survival (≥36 months). (**B**) Comparison of maximum brain metastasis diameter between groups. (**C**) Comparison of total intracranial tumor volume between groups. Individual points represent patients; boxes indicate interquartile ranges (IQRs), and horizontal lines indicate medians. *p* values were calculated using Fisher’s exact test for categorical variables and the Mann–Whitney U test for continuous variables.

To further characterize determinants of durable survival among patients with de novo BM, we compared patients with classifiable extreme survival outcomes (Table 3). Sixteen patients died within 12 months after BM surgery, whereas 16 survived for at least 36 months. Long-term survivors were characterized by a higher prevalence of driver-positive disease and lower intracranial tumor burden. These differences are illustrated in Figure 4.

Driver gene status differed markedly between early-death and long-survival patients. Driver-positive disease was present in 93.8% of long-survival patients, compared with 37.5% of early-death patients (*p* < 0.001). Conversely, no driver-negative patient achieved OS ≥36 months, whereas driver-negative disease accounted for 43.8% of early deaths. EGFR mutations were numerically more frequent among long-term survivors, although this difference did not reach statistical significance.

Intracranial tumor volume burden was also lower among long-term survivors. Maximum BM diameter was smaller in the long-survival group than in the early-death group (median, 2.30 cm vs. 3.30 cm; *p* = 0.026), and total BM volume was lower as well (median, 7.20 vs. 16.65; *p* = 0.028). Log-transformed total BM volume showed the same pattern (*p* = 0.028). In contrast, the number of BM, single versus multiple BM status, BM burden category, and extent of resection did not differ significantly between groups.

Postoperative treatment patterns also differed between groups. All long-term survivors received postoperative systemic therapy, compared with 50.0% of early-death patients (*p* = 0.002), and postoperative radiotherapy was more frequent among long-term survivors (43.8% vs. 18.8%; *p* = 0.027). These treatment-related associations should be interpreted as descriptive findings, because postoperative therapy was not modeled as a time-dependent exposure and may be influenced by treatment opportunity bias. Together, these results suggest that long-term survival after resection is achievable in selected de novo BM patients, particularly those with driver-positive disease and lower intracranial tumor volume burden.

### 3.5. Factors Associated with Long-Term Survival in De Novo BM

Because no driver-negative patient achieved long-term survival in the de novo subgroup, Firth penalized logistic regression was used to reduce small-sample bias and address separation (Table 4). In the model including driver status and maximum BM diameter, driver-negative disease was strongly associated with reduced odds of achieving long-term survival compared with driver-positive disease (OR, 0.046; 95% CI, 0.0003–0.504; *p* = 0.008). Maximum BM diameter showed an adverse but non-significant association after adjustment for driver status (OR per 1 cm increase, 0.581; 95% CI, 0.226–1.273; *p* = 0.176).

A sensitivity model replacing maximum BM diameter with log-transformed total BM volume yielded consistent results. Driver-negative disease remained significantly associated with reduced odds of long-term survival, whereas log-transformed total BM volume showed a non-significant adverse trend. These findings further support a strong association between driver-negative status and a lower likelihood of long-term survival in this exploratory cohort.

In sensitivity analyses using variant-level molecular actionability, non-actionable status remained associated with worse postoperative OS in the multivariable Cox model (HR, 2.16; 95% CI, 1.20–3.90; *p* = 0.010) and with a lower likelihood of long-term survival in the de novo Firth model (OR, 0.063; 95% CI, 0.005–0.437; *p* = 0.004), consistent with the primary analyses based on driver status.

## 4. Discussion

This study provides three clinically relevant observations regarding neurosurgical resection in patients presenting with de novo NSCLC brain metastases. First, de novo presentation itself, though clinically defined as stage IV disease, was not associated with inferior postoperative survival despite a more advanced intracranial disease profile. Second, durable postoperative survival was achievable after resection in selected patients. Third, molecular actionability was strongly associated with durable postoperative survival.

Patients presenting with concurrent lung and brain lesions require individualized decisions regarding tissue acquisition and treatment sequencing, with thoracic biopsy, stereotactic brain biopsy, and neurosurgical resection each having distinct indications. Our findings support the consideration of neurosurgical resection as an initial strategy in appropriately selected patients when surgery is clinically indicated because of neurological symptoms, mass effect, or a surgically accessible dominant lesion. Beyond providing immediate intracranial disease control, resection provides sufficient tissue for comprehensive molecular characterization of the intracranial lesion, which is particularly valuable given the reported molecular discordance between primary lung tumors and brain metastases [19,20]. It also establishes a definitive pathological diagnosis, particularly in the uncommon but clinically important setting of an alternative intracranial malignancy, such as a primary glioma [21,22]. Our study provides real-world evidence to inform multidisciplinary decision-making for patients presenting with concurrent lung and brain lesions. In appropriately selected patients, neurosurgical resection may simultaneously achieve therapeutic and diagnostic objectives through a single procedure, supporting its consideration as an important component of multidisciplinary care rather than solely being a local treatment modality.

Driver status was strongly associated with long-term survival in the exploratory analysis of patients with de novo BM. Although the prevalence of oncogenic driver alterations was comparable between de novo and metachronous disease, long-term survivors within the de novo subgroup were overwhelmingly driver-positive, whereas no driver-negative patient survived beyond 36 months. This likely reflects the transformative impact of CNS-active targeted therapies with improved blood–brain barrier penetration—particularly third-generation EGFR tyrosine kinase inhibitors and newer brain-penetrant agents—while also highlighting the persistent challenges of acquired resistance and durable intracranial disease control [13,14,23].

Beyond molecular status, intracranial tumor burden also appeared to influence long-term outcomes. Consistent with previous reports, our findings suggest that dominant lesion size and total intracranial tumor volume may be more clinically informative than lesion count alone when considering surgical management [24]. Because resection in de novo BM is typically directed toward the symptomatic or dominant lesion rather than all intracranial disease, these measures may better reflect the biological and clinical burden relevant to surgical decision-making. Long-term survivors were also more likely to receive postoperative systemic therapy and radiotherapy, further highlighting the importance of multidisciplinary care [25]. However, these associations should be interpreted descriptively rather than causally, as postoperative treatments were not analyzed as time-dependent variables and may be subject to treatment opportunity bias, whereby patients who survived longer had greater opportunity to receive subsequent therapy.

This study has limitations. It was retrospective and single-center, and the cohort represents patients selected for surgery; therefore, the findings should not be interpreted as evidence supporting the superiority of surgery over non-surgical management. Detailed perioperative morbidity and complication data were not systematically collected, limiting assessment of surgical safety outcomes. Postoperative systemic therapy and radiotherapy were not modeled as time-dependent exposures, so their associations with long-term survival should be interpreted as descriptive rather than causal. The de novo extreme-survival analysis was limited by the small sample size. Molecular data were more frequently unavailable in patients with de novo than metachronous BM, and the reasons for missing molecular testing could not be systematically ascertained retrospectively. Future multicenter studies incorporating non-surgical comparators, molecular treatment sequencing, perioperative functional outcomes, and time-dependent treatment modeling are needed.

## 5. Conclusions

Neurosurgical resection represents a reasonable option for appropriately selected patients with de novo NSCLC-BM, as it may provide immediate intracranial disease control, establish histological diagnosis, and secure tissue for comprehensive molecular profiling. Among surgically treated patients, durable survival was predominantly observed in those with actionable driver alterations and lower intracranial tumor burden, suggesting that molecular actionability and disease burden may be more prognostically relevant than the timing of brain metastasis presentation.

## Figures and Tables

**Table 1:** Baseline characteristics of surgically treated NSCLC-BM patients according to BM timing.

Characteristic	Overall N = 248 ^1^	De Novo N = 113 ^1^	Metachronous N = 135 ^1^	*p*-Value ^2^
age at BM surgery, years	62.00 (55.0, 68.5)	62.00 (55.0, 69.0)	62.00 (56.0, 68.00)	0.539
sex				0.437
female	99 (39.9%)	42 (37.2%)	57 (42.2%)	
male	149 (60.1%)	71 (62.8%)	78 (57.8%)	
smoking status				<0.001
never	137 (55.5%)	57 (50.4%)	80 (59.7%)	
former	54 (21.9%)	11 (9.7%)	43 (32.1%)	
current	56 (22.7%)	45 (39.8%)	11 (8.2%)	
unknown	1	0	1	
weight loss > 5% within 6 months	32 (13.0%)	17 (15.2%)	15 (11.1%)	0.350
unknown	1	1	0	
preoperative KPS category				0.141
≤70	111 (44.8%)	57 (50.4%)	54 (40.0%)	
80	88 (35.5%)	39 (34.5%)	49 (36.3%)	
≥90	49 (19.8%)	17 (15.0%)	32 (23.7%)	
preoperative GCS				0.933
12	2 (0.8%)	1 (0.9%)	1 (0.7%)	
13	5 (2.0%)	3 (2.7%)	2 (1.5%)	
14	3 (1.2%)	1 (0.9%)	2 (1.5%)	
15	238 (96.0%)	108 (95.6%)	130 (96.3%)	
preoperative ECOG category				0.888
0–1	178 (71.8%)	82 (72.6%)	96 (71.1%)	
≥2	70 (28.2%)	31 (27.4%)	39 (28.9%)	
symptomatic BM presentation	203 (81.9%)	105 (92.9%)	98 (72.6%)	<0.001
adenocarcinoma histology	200 (81.3%)	95 (84.8%)	105 (78.4%)	0.250
unknown	2	1	1	
driver gene status				0.005
positive	157 (63.3%)	71 (62.8%)	86 (63.7%)	
negative	72 (29.0%)	27 (23.9%)	45 (33.3%)	
unknown	19 (7.7%)	15 (13.3%)	4 (3.0%)	
EGFR mutation	105 (45.9%)	47 (48.0%)	58 (44.3%)	0.594
unknown	19	15	4	
ALK fusion	16 (7.0%)	8 (8.2%)	8 (6.1%)	0.604
unknown	20	16	4	
KRAS mutation	26 (11.4%)	12 (12.4%)	14 (10.7%)	0.834
unknown	20	16	4	
other rare driver alterations	13 (5.7%)	5 (5.2%)	8 (6.1%)	>0.999
unknown	20	16	4	
number of brain metastases	1.00 (1.00, 3.00)	2.00 (1.00, 4.00)	1.00 (1.00, 1.00)	<0.001
unknown	2	1	1	
brain metastasis number				<0.001
single	150 (61.0%)	49 (43.8%)	101 (75.4%)	
multiple	96 (39.0%)	63 (56.3%)	33 (24.6%)	
unknown	2	1	1	
brain metastasis burden				<0.001
single	150 (61.0%)	49 (43.8%)	101 (75.4%)	
oligo	59 (24.0%)	38 (33.9%)	21 (15.7%)	
multiple	37 (15.0%)	25 (22.3%)	12 (9.0%)	
unknown	2	1	1	
maximum BM diameter, cm	3.10 (2.20, 4.20)	3.00 (2.40, 3.90)	3.30 (2.10, 4.50)	0.634
unknown	3	2	1	
total BM volume	14.88 (5.85, 30.97)	15.09 (7.44, 29.95)	13.67 (3.93, 33.08)	0.328
unknown	3	2	1	
log-transformed total BM volume	2.77 (1.92, 3.46)	2.78 (2.13, 3.43)	2.68 (1.60, 3.53)	0.328
unknown	3	2	1	
location of brain metastases				<0.001
supratentorial	172 (70.2%)	64 (57.1%)	108 (81.2%)	
infratentorial	20 (8.2%)	8 (7.1%)	12 (9.0%)	
both	53 (21.6%)	40 (35.7%)	13 (9.8%)	
unknown	3	1	2	
leptomeningeal disease	40 (16.2%)	16 (14.3%)	24 (17.8%)	0.492
unknown	1	1	0	
eloquent area involvement	119 (48.2%)	60 (53.6%)	59 (43.7%)	0.127
unknown	1	1	0	
extracranial disease status at surgery				0.021
none	127 (51.4%)	47 (42.0%)	80 (59.3%)	
stable	60 (24.3%)	31 (27.7%)	29 (21.5%)	
progressive	60 (24.3%)	34 (30.4%)	26 (19.3%)	
unknown	1	1	0	
combined intracranial–extracranial disease extent				<0.001
single BM only	91 (36.8%)	28 (25.0%)	63 (46.7%)	
single BM plus systemic	64 (25.9%)	22 (19.6%)	42 (31.1%)	
multiple BM	92 (37.2%)	62 (55.4%)	30 (22.2%)	
unknown	1	1	0	
preoperative systemic therapy	126 (51.0%)	13 (11.5%)	113 (84.3%)	<0.001
unknown	1	0	1	
extent of resection, binary				<0.001
GTR	136 (55.1%)	43 (38.4%)	93 (68.9%)	
non-GTR	111 (44.9%)	69 (61.6%)	42 (31.1%)	
unknown	1	1	0	
extent of resection				<0.001
GTR	136 (55.1%)	43 (38.4%)	93 (68.9%)	
STR	31 (12.6%)	16 (14.3%)	15 (11.1%)	
PR	80 (32.4%)	53 (47.3%)	27 (20.0%)	
unknown	1	1	0	
RPA class				0.595
I	132 (53.4%)	57 (50.9%)	75 (55.6%)	
II	73 (29.6%)	33 (29.5%)	40 (29.6%)	
III	42 (17.0%)	22 (19.6%)	20 (14.8%)	
unknown	1	1	0	
GPA category				<0.001
≤1	35 (14.2%)	25 (22.3%)	10 (7.4%)	
>1	212 (85.8%)	87 (77.7%)	125 (92.6%)	
unknown	1	1	0	

^1^ Median (Q1, Q3); n (%). ^2^ Wilcoxon rank sum test; Fisher’s exact test; Fisher’s exact test for count data with simulated *p*-value. Abbreviations: ALK, anaplastic lymphoma kinase; BM, brain metastasis; ECOG, Eastern Cooperative Oncology Group; EGFR, epidermal growth factor receptor; GPA, Graded Prognostic Assessment; GCS, Glasgow Coma Scale; GTR, gross total resection; KPS, Karnofsky Performance Status; NSCLC, non-small-cell lung cancer; PR, partial resection; RPA, recursive partitioning analysis; STR, subtotal resection.

**Table 2:** Multivariable Cox regression analysis for overall survival in the OS-evaluable cohort.

Characteristic	HR	95% CI	*p*-Value
BM			
de novo	—	—	
metachronous	0.96	0.51, 1.81	>0.9
driver gene status			
positive	—	—	
negative	3.31	1.81, 6.06	<0.001
unknown	3.27	1.21, 8.79	0.019
maximum BM diameter, per cm	1.37	1.12, 1.69	0.003
combined intracranial–extracranial disease extent			
single BM only	—	—	
single BM plus systemic	2.97	1.44, 6.12	0.003
multiple BM	1.95	0.95, 4.02	0.071
weight loss > 5% within 6 months			
no	—	—	
yes	1.79	0.90, 3.54	0.10

Abbreviations: CI = Confidence Interval, HR = Hazard Ratio. HRs were estimated using a multivariable Cox proportional hazards model including BM timing, driver gene status, maximum BM diameter, combined intracranial–extracranial disease extent, and recent weight loss.

**Table 3:** Characteristics of de novo NSCLC-BM patients with early death and long-term survival after surgical resection.

Characteristic	Overall N = 32	Early Death (<12 Months) N = 16	Long-Term Survival (≥36 Months) N = 16	*p* Value
age at BM surgery, years	64.50 (56.00, 70.50)	66.50 (61.50, 70.50)	62.00 (54.50, 70.50)	0.533
sex				0.285
female	14 (43.8%)	5 (31.3%)	9 (56.3%)	
male	18 (56.3%)	11 (68.8%)	7 (43.8%)	
smoking status				0.030
never	18 (56.3%)	6 (37.5%)	12 (75.0%)	
former	3 (9.4%)	1 (6.3%)	2 (12.5%)	
current	11 (34.4%)	9 (56.3%)	2 (12.5%)	
weight loss > 5% within 6 months	3 (9.4%)	3 (18.8%)	0 (0.0%)	0.226
preoperative KPS category				0.275
≤70	15 (46.9%)	9 (56.3%)	6 (37.5%)	
80	11 (34.4%)	6 (37.5%)	5 (31.3%)	
≥90	6 (18.8%)	1 (6.3%)	5 (31.3%)	
preoperative GCS				>0.999
13	2 (6.3%)	1 (6.3%)	1 (6.3%)	
15	30 (93.8%)	15 (93.8%)	15 (93.8%)	
preoperative ECOG category				0.473
0–1	19 (59.4%)	8 (50.0%)	11 (68.8%)	
≥2	13 (40.6%)	8 (50.0%)	5 (31.3%)	
symptomatic BM presentation	29 (90.6%)	15 (93.8%)	14 (87.5%)	>0.999
adenocarcinoma histology	29 (90.6%)	13 (81.3%)	16 (100.0%)	0.226
driver gene status				<0.001
positive	21 (65.6%)	6 (37.5%)	15 (93.8%)	
negative	7 (21.9%)	7 (43.8%)	0 (0.0%)	
unknown	4 (12.5%)	3 (18.8%)	1 (6.3%)	
EGFR mutation	14 (50.0%)	4 (30.8%)	10 (66.7%)	0.128
unknown	4	3	1	
ALK fusion	2 (7.4%)	0 (0.0%)	2 (13.3%)	0.487
unknown	5	4	1	
KRAS mutation	2 (7.4%)	1 (8.3%)	1 (6.7%)	>0.999
unknown	5	4	1	
other rare driver alterations	3 (11.1%)	1 (8.3%)	2 (13.3%)	>0.999
unknown	5	4	1	
number of brain metastases	2.00 (1.00, 5.00)	1.00 (1.00, 4.00)	2.50 (1.00, 5.50)	0.241
unknown	1	1	0	
brain metastasis number				0.285
single	13 (41.9%)	8 (53.3%)	5 (31.3%)	
multiple	18 (58.1%)	7 (46.7%)	11 (68.8%)	
unknown	1	1	0	
brain metastasis burden				0.480
single	13 (41.9%)	8 (53.3%)	5 (31.3%)	
oligo	10 (32.3%)	4 (26.7%)	6 (37.5%)	
multiple	8 (25.8%)	3 (20.0%)	5 (31.3%)	
unknown	1	1	0	
maximum BM diameter, cm	2.80 (2.00, 3.70)	3.30 (2.60, 4.40)	2.30 (1.90, 3.00)	0.026
unknown	2	1	1	
total BM volume	14.00 (5.70, 22.71)	16.65 (7.44, 30.80)	7.20 (2.16, 17.36)	0.028
unknown	2	1	1	
log-transformed total BM volume	2.71 (1.90, 3.17)	2.87 (2.13, 3.46)	2.10 (1.15, 2.91)	0.028
unknown	2	1	1	
location of brain metastases				0.112
supratentorial	18 (58.1%)	9 (60.0%)	9 (56.3%)	
infratentorial	3 (9.7%)	3 (20.0%)	0 (0.0%)	
both	10 (32.3%)	3 (20.0%)	7 (43.8%)	
unknown	1	1	0	
leptomeningeal disease	6 (19.4%)	5 (33.3%)	1 (6.3%)	0.083
unknown	1	1	0	
eloquent area involvement	16 (51.6%)	8 (53.3%)	8 (50.0%)	>0.999
unknown	1	1	0	
extracranial disease status at surgery				0.349
none	12 (38.7%)	7 (46.7%)	5 (31.3%)	
stable	8 (25.8%)	2 (13.3%)	6 (37.5%)	
progressive	11 (35.5%)	6 (40.0%)	5 (31.3%)	
unknown	1	1	0	
combined intracranial–extracranial disease extent				0.334
single BM only	6 (19.4%)	3 (20.0%)	3 (18.8%)	
single BM plus systemic	7 (22.6%)	5 (33.3%)	2 (12.5%)	
multiple BM	18 (58.1%)	7 (46.7%)	11 (68.8%)	
unknown	1	1	0	
preoperative systemic therapy	4 (12.5%)	3 (18.8%)	1 (6.3%)	0.600
extent of resection (binary)				>0.999
GTR	10 (32.3%)	5 (33.3%)	5 (31.3%)	
non-GTR	21 (67.7%)	10 (66.7%)	11 (68.8%)	
unknown	1	1	0	
extent of resection				>0.999
GTR	10 (32.3%)	5 (33.3%)	5 (31.3%)	
STR	6 (19.4%)	3 (20.0%)	3 (18.8%)	
PR	15 (48.4%)	7 (46.7%)	8 (50.0%)	
unknown	1	1	0	
RPA class				0.261
I	13 (41.9%)	4 (26.7%)	9 (56.3%)	
II	8 (25.8%)	5 (33.3%)	3 (18.8%)	
III	10 (32.3%)	6 (40.0%)	4 (25.0%)	
unknown	1	1	0	
GPA category				0.433
≤1	8 (25.8%)	5 (33.3%)	3 (18.8%)	
>1	23 (74.2%)	10 (66.7%)	13 (81.3%)	
unknown	1	1	0	
postoperative systemic therapy				0.002
yes	24 (75.0%)	8 (50.0%)	16 (100.0%)	
no	4 (12.5%)	4 (25.0%)	0 (0.0%)	
missing	4 (12.5%)	4 (25.0%)	0 (0.0%)	
postoperative radiotherapy				0.027
yes	10 (31.3%)	3 (18.8%)	7 (43.8%)	
no	16 (50.0%)	7 (43.8%)	9 (56.3%)	
missing	6 (18.8%)	6 (37.5%)	0 (0.0%)	

**Table 4:** Firth penalized logistic regression for long-term survival in de novo BM.

Characteristic	OR	95% CI	*p*-Value
driver gene status			
positive	reference	—	—
negative	0.046	0.0003–0.504	0.008
maximum BM diameter (per cm)	0.581	0.226–1.273	0.176

Outcome: long-term survival (OS ≥ 36 months) versus early death (<12 months) after BM surgery.

## Data Availability

The datasets analyzed during the current study are not publicly available due to patient privacy and institutional regulations, but are available from the corresponding authors upon reasonable request.

## Supplementary Materials

The following supporting information can be downloaded at: https://www.mdpi.com/article/10.3390/jcm15186955/s1, Table S1. Baseline characteristics of the OS-evaluable cohort according to BM timing.

## References

[1] Le Rhun E., Guckenberger M., Smits M., Dummer R., Bachelot T., Sahm F., Galldiks N., de Azambuja E., Berghoff A.S., Metellus P. (2021). EANO-ESMO Clinical Practice Guidelines for diagnosis, treatment and follow-up of patients with brain metastasis from solid tumours. Ann. Oncol..

[2] Vogelbaum M.A., Brown P.D., Messersmith H., Brastianos P.K., Burri S., Cahill D., Dunn I.F., Gaspar L.E., Gatson N.T.N., Gondi V. (2022). Treatment for Brain Metastases: ASCO-SNO-ASTRO Guideline. J. Clin. Oncol..

[3] Agazzi S., Pampallona S., Pica A., Vernet O., Regli L., Porchet F., Villemure J.G., Leyvraz S. (2004). The origin of brain metastases in patients with an undiagnosed primary tumour. Acta Neurochir..

[4] Sankey E.W., Tsvankin V., Grabowski M.M., Nayar G., Batich K.A., Risman A., Champion C.D., Salama A.K., Goodwin C.R., Fecci P.E. (2019). Operative and peri-operative considerations in the management of brain metastasis. Cancer Med..

[5] Yu K.K.H., Patel A.R., Moss N.S. (2020). The Role of Stereotactic Biopsy in Brain Metastases. Neurosurg. Clin. N. Am..

[6] Soffietti R., Abacioglu U., Baumert B., Combs S.E., Kinhult S., Kros J.M., Marosi C., Metellus P., Radbruch A., Villa Freixa S.S. (2017). Diagnosis and treatment of brain metastases from solid tumors: Guidelines from the European Association of Neuro-Oncology (EANO). Neuro Oncol..

[7] Karschnia P., Le Rhun E., Vogelbaum M.A., Van den Bent M., Grau S.J., Preusser M., Soffietti R., von Baumgarten L., Westphal M., Weller M. (2021). The evolving role of neurosurgery for central nervous system metastases in the era of personalized cancer therapy. Eur. J. Cancer.

[8] Kahraman S., Karakaya S., Kaplan M.A., Goksu S.S., Ozturk A., Isleyen Z.S., Hamdard J., Yildirim S., Dogan T., Isik S. (2024). Treatment outcomes and prognostic factors in patients with driver mutant non-small cell lung cancer and de novo brain metastases. Sci. Rep..

[9] Soffietti R., Ahluwalia M., Lin N., Rudà R. (2020). Management of brain metastases according to molecular subtypes. Nat. Rev. Neurol..

[10] Solomon B.J., Bauer T.M., Ignatius Ou S.H., Liu G., Hayashi H., Bearz A., Penkov K., Wu Y.L., Arrieta O., Jassem J. (2022). Post Hoc Analysis of Lorlatinib Intracranial Efficacy and Safety in Patients With ALK-Positive Advanced Non-Small-Cell Lung Cancer From the Phase III CROWN Study. J. Clin. Oncol..

[11] Zhao Y., Li S., Yang X., Chu L., Wang S., Tong T., Chu X., Yu F., Zeng Y., Guo T. (2022). Overall survival benefit of osimertinib and clinical value of upfront cranial local therapy in untreated EGFR-mutant nonsmall cell lung cancer with brain metastasis. Int. J. Cancer.

[12] Ahluwalia M.S., Becker K., Levy B.P. (2018). Epidermal Growth Factor Receptor Tyrosine Kinase Inhibitors for Central Nervous System Metastases from Non-Small Cell Lung Cancer. Oncologist.

[13] Wang X., Chi Y., Mu F., Mao H., Yang C., Yang M. (2026). Targeting MET in non-small cell lung cancer brain metastases: From molecular mechanism to precision therapy. J. Transl. Med..

[14] Wang B., Guo H., Xu H., Yu H., Chen Y., Zhao G. (2021). Research Progress and Challenges in the Treatment of Central Nervous System Metastasis of Non-Small Cell Lung Cancer. Cells.

[15] Peters S., Camidge R., Dziadziuszko R., Gadgeel S., Shaw A.T., Kim D.W., Pérol M., Rosell D.R., Cheema P., Lim D.W.T. (2026). Alectinib versus crizotinib in previously untreated ALK-positive advanced non-small cell lung cancer: Final overall survival analysis of the phase III ALEX study. Ann. Oncol..

[16] Weller M., Remon J., Rieken S., Vollmuth P., Ahn M.J., Minniti G., Le Rhun E., Westphal M., Brastianos P.K., Soo R.A. (2024). Central nervous system metastases in advanced non-small cell lung cancer: A review of the therapeutic landscape. Cancer Treat. Rev..

[17] Hendriks L.E., Henon C., Auclin E., Mezquita L., Ferrara R., Audigier-Valette C., Mazieres J., Lefebvre C., Rabeau A., Le Moulec S. (2019). Outcome of Patients with Non-Small Cell Lung Cancer and Brain Metastases Treated with Checkpoint Inhibitors. J. Thorac. Oncol..

[18] Billing P.S., Miller D.L., Allen M.S., Deschamps C., Trastek V.F., Pairolero P.C. (2001). Surgical treatment of primary lung cancer with synchronous brain metastases. J. Thorac. Cardiovasc. Surg..

[19] Matsumoto S., Takahashi K., Iwakawa R., Matsuno Y., Nakanishi Y., Kohno T., Shimizu E., Yokota J. (2006). Frequent EGFR mutations in brain metastases of lung adenocarcinoma. Int. J. Cancer.

[20] Palmer J.D., Trifiletti D.M., Gondi V., Chan M., Minniti G., Rusthoven C.G., Schild S.E., Mishra M.V., Bovi J., Williams N. (2020). Multidisciplinary patient-centered management of brain metastases and future directions. Neuro Oncol. Adv..

[21] Carmicheal J., Johnson K.C., Neilsen B.K., Shonka N., Zhang C., Baine M. (2025). Synchronous Double Primary Malignancy of Non-Small Cell Lung Cancer and Glioblastoma: A Case Report and Literature Review. Case Rep. Oncol..

[22] Shahsavari N., Ahmad M., Sekar V., Meola A., Hancock S.L., Chang S.D., Chiang V.L. (2022). Synchronous glioblastoma and brain metastases: Illustrative case. J. Neurosurg. Case Lessons.

[23] Rotow J., Bivona T.G. (2017). Understanding and targeting resistance mechanisms in NSCLC. Nat. Rev. Cancer.

[24] Yang K., Gutiérrez-Valencia E., Landry A.P., Kalyvas A., Millesi M., Leite M., Jablonska P.A., Weiss J., Millar B.A., Conrad T. (2022). Multiplicity does not significantly affect outcomes in brain metastasis patients treated with surgery. Neurooncol. Adv..

[25] Moss N.S., Beal K., Tabar V. (2022). Brain Metastasis-A Distinct Oncologic Disease Best Served by an Integrated Multidisciplinary Team Approach. JAMA Oncol..

