# Supplementary material for: Neurosurgical Resection for De Novo Brain Metastases from Non-Small Cell Lung Cancer: Real-World Evidence for Multidisciplinary Decision-Making
Source: J Clin Med. 2026 Sep 8;15(18):6955. doi: 10.3390/jcm15186955 (PMC13607701; doi:10.3390/jcm15186955)
Supplement: Supplementary file 1 [file jcm-15-06955-s001.zip › jcm-4504677-supplementary.pdf]

Supplementary Table S1. Baseline characteristics of the OS-evaluable cohort according to BM timing

Characteristic	Overall N = 161[1]	de novo N = 73[1]	metachronous N = 88[1]	p-value[2]
**Age at BM surgery, years**	63.00 (56.00, 69.00)	62.00 (55.00, 69.00)	63.00 (57.00, 68.50)	0.930
**Sex**				0.872
female	66 (41.0%)	29 (39.7%)	37 (42.0%)	
male	95 (59.0%)	44 (60.3%)	51 (58.0%)	
**Smoking status**				<0.001
never	91 (56.5%)	40 (54.8%)	51 (58.0%)	
former	35 (21.7%)	6 (8.2%)	29 (33.0%)	
current	35 (21.7%)	27 (37.0%)	8 (9.1%)	
**Weight loss >5% within 6 months**	20 (12.4%)	11 (15.1%)	9 (10.2%)	0.472
**Preoperative KPS**				0.164
40	2 (1.2%)	2 (2.7%)	0 (0.0%)	
50	11 (6.8%)	5 (6.8%)	6 (6.8%)	
60	16 (9.9%)	11 (15.1%)	5 (5.7%)	
70	43 (26.7%)	19 (26.0%)	24 (27.3%)	
80	51 (31.7%)	24 (32.9%)	27 (30.7%)	
90	33 (20.5%)	10 (13.7%)	23 (26.1%)	
100	5 (3.1%)	2 (2.7%)	3 (3.4%)	
**Preoperative KPS category**				0.145
≤70	72 (44.7%)	37 (50.7%)	35 (39.8%)	
80	51 (31.7%)	24 (32.9%)	27 (30.7%)	
≥90	38 (23.6%)	12 (16.4%)	26 (29.5%)	
**Preoperative GCS**				0.924
12	1 (0.6%)	0 (0.0%)	1 (1.1%)	
13	5 (3.1%)	3 (4.1%)	2 (2.3%)	
14	3 (1.9%)	1 (1.4%)	2 (2.3%)	
15	152 (94.4%)	69 (94.5%)	83 (94.3%)	
**Preoperative ECOG**				0.221

Characteristic	Overall<br>N = 161[1]	de novo<br>N = 73[1]	metachronous<br>N = 88[1]	p-value[2]
0	31 (19.3%)	9 (12.3%)	22 (25.0%)	
1	82 (50.9%)	41 (56.2%)	41 (46.6%)	
2	39 (24.2%)	18 (24.7%)	21 (23.9%)	
3	9 (5.6%)	5 (6.8%)	4 (4.5%)	
**Preoperative ECOG category**				0.730
0-1	113 (70.2%)	50 (68.5%)	63 (71.6%)	
≥2	48 (29.8%)	23 (31.5%)	25 (28.4%)	
**Symptomatic BM presentation**	126 (78.3%)	65 (89.0%)	61 (69.3%)	0.004
**Adenocarcinoma histology**	134 (83.2%)	66 (90.4%)	68 (77.3%)	0.034
**Driver gene status**				0.050
positive	108 (67.1%)	49 (67.1%)	59 (67.0%)	
negative	43 (26.7%)	16 (21.9%)	27 (30.7%)	
unknown	10 (6.2%)	8 (11.0%)	2 (2.3%)	
**EGFR mutation**	72 (47.7%)	33 (50.8%)	39 (45.3%)	0.516
Unknown	10	8	2	
**ALK fusion**	10 (6.7%)	6 (9.4%)	4 (4.7%)	0.326
Unknown	11	9	2	
**KRAS mutation**	18 (12.0%)	6 (9.4%)	12 (14.0%)	0.454
Unknown	11	9	2	
**Other rare driver alterations**	10 (6.7%)	5 (7.8%)	5 (5.8%)	0.745
Unknown	11	9	2	
**Number of brain metastases**	1.00 (1.00, 2.00)	2.00 (1.00, 4.00)	1.00 (1.00, 1.00)	<0.001
Unknown	1	1	0	
**Brain metastasis number**				<0.001
single	102 (63.8%)	32 (44.4%)	70 (79.5%)	
multiple	58 (36.3%)	40 (55.6%)	18 (20.5%)	
Unknown	1	1	0	
**Brain metastasis burden**				<0.001
single	102 (63.8%)	32 (44.4%)	70 (79.5%)	

Characteristic	Overall<br>N = 161[1]	de novo<br>N = 73[1]	metachronous<br>N = 88[1]	p-value[2]
oligo	35 (21.9%)	23 (31.9%)	12 (13.6%)	
multiple	23 (14.4%)	17 (23.6%)	6 (6.8%)	
Unknown	1	1	0	
**Maximum BM diameter, cm**	3.10 (2.20, 4.10)	3.00 (2.20, 3.80)	3.30 (2.05, 4.40)	0.908
Unknown	2	2	0	
**Total BM volume**	15.05 (5.20, 28.81)	16.20 (7.20, 27.41)	11.95 (3.41, 29.33)	0.359
Unknown	2	2	0	
**Log-transformed total BM volume**	2.78 (1.82, 3.39)	2.84 (2.10, 3.35)	2.56 (1.48, 3.41)	0.359
Unknown	2	2	0	
**Location of brain metastases**				<0.001
supratentorial	113 (71.1%)	43 (59.7%)	70 (80.5%)	
infratentorial	12 (7.5%)	3 (4.2%)	9 (10.3%)	
both	34 (21.4%)	26 (36.1%)	8 (9.2%)	
Unknown	2	1	1	
**Leptomeningeal disease**	26 (16.3%)	12 (16.7%)	14 (15.9%)	>0.999
Unknown	1	1	0	
**Eloquent area involvement**	79 (49.4%)	41 (56.9%)	38 (43.2%)	0.112
Unknown	1	1	0	
**Extracranial disease status at surgery**				0.010
none	84 (52.5%)	29 (40.3%)	55 (62.5%)	
stable	37 (23.1%)	23 (31.9%)	14 (15.9%)	
progressive	39 (24.4%)	20 (27.8%)	19 (21.6%)	
Unknown	1	1	0	
**Combined intracranial-extracranial disease extent**				<0.001
Single BM only	64 (40.0%)	18 (25.0%)	46 (52.3%)	
Single BM plus systemic	40 (25.0%)	14 (19.4%)	26 (29.5%)	

Characteristic	Overall N = 161[1]	de novo N = 73[1]	metachronous N = 88[1]	p-value[2]
Multiple BM	56 (35.0%)	40 (55.6%)	16 (18.2%)	
Unknown	1	1	0	
**Preoperative systemic therapy**	82 (50.9%)	7 (9.6%)	75 (85.2%)	<0.001
**Extent of resection, binary**				<0.001
GTR	95 (59.4%)	27 (37.5%)	68 (77.3%)	
non-GTR	65 (40.6%)	45 (62.5%)	20 (22.7%)	
Unknown	1	1	0	
**Extent of resection**				<0.001
GTR	95 (59.4%)	27 (37.5%)	68 (77.3%)	
STR	18 (11.3%)	8 (11.1%)	10 (11.4%)	
PR	47 (29.4%)	37 (51.4%)	10 (11.4%)	
Unknown	1	1	0	
**RPA class**				0.065
I	84 (52.5%)	36 (50.0%)	48 (54.5%)	
II	50 (31.3%)	19 (26.4%)	31 (35.2%)	
III	26 (16.3%)	17 (23.6%)	9 (10.2%)	
Unknown	1	1	0	
**GPA score**				<0.001
0	1 (0.6%)	1 (1.4%)	0 (0.0%)	
0.5	6 (3.8%)	5 (6.9%)	1 (1.1%)	
1	17 (10.6%)	12 (16.7%)	5 (5.7%)	
1.5	18 (11.3%)	14 (19.4%)	4 (4.5%)	
2	33 (20.6%)	14 (19.4%)	19 (21.6%)	
2.5	26 (16.3%)	8 (11.1%)	18 (20.5%)	
3	26 (16.3%)	7 (9.7%)	19 (21.6%)	
3.5	22 (13.8%)	6 (8.3%)	16 (18.2%)	
4	11 (6.9%)	5 (6.9%)	6 (6.8%)	
Unknown	1	1	0	
**GPA category**				0.002

Characteristic	Overall N = 161[1]	de novo N = 73[1]	metachronous N = 88[1]	p-value[2]
≤1	24 (15.0%)	18 (25.0%)	6 (6.8%)	
>1	136 (85.0%)	54 (75.0%)	82 (93.2%)	
Unknown	1	1	0	

[1]Median (Q1, Q3); n (%)

[2]Wilcoxon rank sum test; Fisher's exact test; Fisher's Exact Test for Count Data with simulated p-value (based on 20000 replicates)

Abbreviations: ALK, anaplastic lymphoma kinase; BM, brain metastasis; ECOG, Eastern Cooperative Oncology Group; EGFR, epidermal growth factor receptor; GPA, Graded Prognostic Assessment; GCS, Glasgow Coma Scale; GTR, gross total resection; KPS, Karnofsky Performance Status; NSCLC, non–small cell lung cancer; PR, partial resection; RPA, recursive partitioning analysis; STR, subtotal resection.
